# Comparison of Characteristics and Survival of New-Onset Hepatocellular Carcinomas With or Without Cirrhosis in a Tertiary Center

**DOI:** 10.5152/tjg.2024.23451

**Published:** 2024-07-01

**Authors:** Filiz Araz, Barış Soydaş, Birol Özer, Elif Karadeli, Gürcan Erbay

**Affiliations:** 1 Department of Gastroenterology, Başkent University, Adana Turgut Noyan Training and Research Hospital, Adana, Türkiye; 2 Department of Radiology, Başkent University, Adana Turgut Noyan Training and Research Hospital, Adana, Türkiye

**Keywords:** Hepatocellular carcinoma, cirrhosis, survival

## Abstract

**Background/Aims:**

Although hepatocellular carcinoma (HCC) usually develops in cirrhotic livers, HCCs could also arise in non-cirrhotic livers. We aimed to compare the characteristics and survival of cirrhotic- and non-cirrhotic HCCs.

**Materials and Methods:**

Data of HCC patients between 2011 and 2021 in a single tertiary center was evaluated retrospectively. Demographic, clinical, laboratory, tumoral and pathological features, and survival outcomes of cirrhotic and non-cirrhotic HCCs were compared.

**Results:**

Thestudy included 188 HCC patients. Median age was 64 (26-92) years and similar for study groups (*P* = .208). Both groups had similar male/female ratio. Forty-two patients (22.3%) had HCC in non-cirrhotic liver. Non-cirrhotic HCCs had similar tumor differentiation type, radiological characteristics, Milan, University of California San Francisco, and the Barcelona Clinic Liver Cancer stages, but more unifocal lesion (78.6% vs. 59.6%) and larger tumor size (89.5 (16-240) mm vs. 59.0 (12-290) mm) at presentation compared to non-cirrhotic HCCs. Despite larger tumor size, non-cirrhotic HCC patients had better overall, disease-free and progression-free survival rates than cirrhotic HCCs. Overall survivals for 1 and 3 years were 71.4% and 49.7% for non-cirrhotic and 54% and 28.3% for cirrhotic HCCs, respectively (*P* = .035). According to Cox analyses, Eastern Cooperative Oncology Group score (*P* < .001, hazards ratio (HR): 4.05) and curative treatments (*P* < .001, HR: 0.21) were predictive for overall survival in cirrhotic HCCs. Curative treatment (*P* = .027, HR: 0.31) was found to be a significant predictor for overall survival in non-cirrhotic HCCs. Vascular invasion was the only independent predictor for disease-free survival (HR: 2.62, 95% CI 1.01-6.93, *P* = .049) for non-cirrhotic HCCs.

**Conclusion:**

Despite larger tumor size and similar tumor stages, compared to cirrhotic HCCs, non-cirrhotic HCCs were associated with better survival outcomes.

**Main Points**

- Hepatocellular carcinomas (HCCs) develop in non-cirrhotic basis with considerable frequency.
- Compared to cirrhotic HCCs, non-cirrhotic patients have more unifocal and larger HCCs with similar pathological differentiation, age, and gender distribution.
- There are better overall, disease-free, and progression-free survival rates for HCCs without cirrhosis compared to those with cirrhosis.

## Introduction

Hepatocellular carcinoma (HCC) is the sixth most common cause of cancer worldwide. Hepatocellular carcinoma ranks third order as a cause for global cancer-related mortality.^1^ The most commonly associated risk factors are hepatitis B virus (HBV) infection, hepatitis C virus (HCV) infection, alcohol, and non-alcoholic fatty liver disease (NAFLD).^2^ Non-alcoholic fatty liver disease is a fast-growing reason for chronic liver disease and the main contributor to the burden of HCC, not only in Western countries but all over the world.^3,4^

Hepatocellular carcinoma arises primarily in the setting of chronic liver disease and cause significant mortality among this group.^5^ However, HCC may also develop in the liver with no cirrhosis. Underlying mechanisms for HCC development among patients with no cirrhosis are not clear. A range of 15%-20% of HCCs were diagnosed in the absence of cirrhosis in Western countries.^6,7^ In a recent report, 20% of NAFLD patients with HCC had no underlying cirrhosis.^8^ Non-alcoholic fatty liver disease and metabolic syndrome are the most common etiologies linked to non-cirrhotic HCC in the US, ^9,10^ while viral hepatitis and alcohol are more frequently reported etiologies in Europe and Asia.^11-14^

Hepatocellular carcinomas in non-cirrhotic patients have been reported to be diagnosed at more advanced tumor stages than HCC in cirrhotic patients.^11,15^ Surveillance ultrasound examinations, which are recommended for cirrhotic patients with significant risk, may be an explanation for this difference. However, curative treatments for HCC in cirrhotic patients can only be suitable for a small subset of patients. More HCC patients with non-cirrhotic liver could be candidates for curative surgical interventions without significant postoperative decompensation risk due to the preserved hepatic functions.^12^

We aimed to define clinical and radiological characteristics of new onset HCCs developing on cirrhotic and non-cirrhotic liver and to compare survival outcomes of these groups.

## Materials and Methods

Data of patients with new diagnosis of HCC by radiology or pathology in Başkent University Adana Dr. Turgut Noyan Training and Research Hospital between January 2011 and December 2021 were reviewed retrospectively. The study was approved by the Institutional Review Board of Başkent University (approval no: KA21/430 date: November 5, 2021). Hepatocellular carcinoma patients <18 years old who were diagnosed prior to 2011, had metastatic liver disease, cholangiocarcinoma or combined HCC/cholangiocarcinoma, hepatoblastoma, HCC recurrence, or indefinite diagnosis, or had incomplete data to define the status of cirrhosis at diagnosis were excluded. Informed consent was not taken from the patients for this retrospective study.

Demographic findings, presence of cirrhosis, tumor number, total and maximal tumor diameter, stage of cirrhosis (if present), alpha-fetoprotein level (AFP), laboratory findings, underlying etiology, radiological characteristics on diagnosis, and treatment modalities applied for HCC were recorded. All patients had at least 1 dynamic liver imaging, either magnetic resonance imaging (MRI) or computed tomography (CT). Positron emission tomography (PET) findings were recorded, if available. Disease-free, progression-free, and overall survival (OS) periods were defined for all patients.

Cirrhosis was assessed mainly by histology. If liver parenchymal biopsy is not available, radiological findings, presence of varices and/or splenomegaly were used for the diagnosis of cirrhosis. Criteria proposed by Mittal et al^9^ were applied to classify the status of cirrhosis. Liver cirrhosis was considered to be present if the patient has clinical features of portal hypertension including ascites, varices, or hepatic encephalopathy, or has pathological findings compatible with cirrhosis, or has 2 of the following 3 laboratory findings: albumin < 3 gr/L, platelets < 200 000 /mL, international normalized ratio (INR) > 1.1 within a period of 6 months before and 4 weeks after the diagnosis of HCC. Patients without cirrhosis were categorized as level 1 and level 2 evidence for the absence of cirrhosis according to Mittal et al.^9^ For level 1 evidence for the absence of cirrhosis, there should be no histological evidence for cirrhosis within biopsies taken at the time or 1 year before the diagnosis of HCC without compatible cirrhotic features on radiology applied nearest to the diagnosis in the past 3 years before the HCC diagnosis. Level 2 evidence for the absence of cirrhosis requires the presence of APRI lower than 1, and 2 of 3 laboratory findings within the normal range (albumin > 3.5 g/L, platelets > 200 000/mL, INR < 1.1), on the basis of laboratory data available nearest to the HCC diagnosis (in the range of 6 months before and 4 weeks after the diagnosis) together with no compatible radiologic findings for cirrhosis present nearest to the HCC diagnosis (within a range of 3 years before the diagnosis).

Hepatocellular carcinoma was diagnosed mainly by biopsy findings. In the absence of histological findings, HCC was diagnosed according to Liver Imaging Reporting and Data System 5 criteria.^16^

Child-Pugh-Turcotte (CPT) and Model For End-Stage Liver Disease scores were calculated accordingly for cirrhotic patients with HCC. Milan, University of California San Francisco (UCSF), and the Barcelona Clinic Liver Cancer (BCLC) stages were defined for all patients on initial diagnosis. Milan criteria are defined as unifocal tumor diameter less than 5 cm or 3 or fewer tumors each less than 3 cm in diameter, without vascular invasion or extrahepatic disease.^17^ Criteria of USCF necessitate a single tumor equal to or less than 6.5 cm in diameter or three or less tumors with the maximal tumor diameter of ≤4.5 cm and total tumor diameter ≤8 cm, without vascular involvement or extrahepatic disease.^18^ Clinical, radiologic, and laboratory data were used to assign BCLC stage (stage 0, A, B, C, and D).^19^

Treatments including liver transplantation, resection, and radiofrequency ablation (RFA) were considered as curative treatments. Palliative treatments included trans-arterial chemoembolization, trans-arterial radioembolization, and systemic therapies.

### Survival Status

Patient survival was figured out from the database. In the case of alive patient, the last available contact date in the database was used to define the censoring time for the survival analysis. Overall survival defines the time starting from the HCC diagnosis to the date of death according to the hospital records and/or the national death notification system, or censored on December 31, 2021, if alive. Disease-free survival (DFS) defines the period from HCC diagnosis to the date of recurrence. Progression-free survival (PFS) includes the time period from the HCC diagnosis to the date of progression, censored at the date of the last contact.

### Statistical Analysis

Chi-square tests were applied for comparison of categorical data and the Mann–Whitney *U*-test was used for testing independent samples in continuous data. The survival probability was estimated by the Kaplan–Meier method and compared between groups using the log‐rank test. Cox proportional hazards regression models were used to evaluate the association between the risk factors and the OS, DFS, and PFS. Variables that were significant at *P* < .10 in the univariate analysis were included in a stepwise selection procedure to select which variables would be included in the final Cox multivariate regression model. Variables of clinical significance including age, sex, platelets, and underlying etiology were always retained in the model. Results for numerical data are reported as medians with IQR for continuous variables and as numbers and percentages for categorical variables. *P* < .05 was considered statistically significant. All tests were carried out two-sided. All statistical analyses were performed using the Statistical Package for the Social Sciences Version 20.0 (IBM Corp., Armonk, NY, USA).

## Results

### General Characteristics of Non-cirrhotic and Cirrhotic HCC Patients

A total of 188 patients were included. Median age was 64 (26-92) years, and 154 (81.9%) patients were male. Hepatitis B and C were present in 89 (47.3%) and 39 (20.7%) patients, respectively. Alcoholic liver cirrhosis was present in 10 (5.3%) cases. No underlying disease was present in 50 (26.6%) patients. Dynamic CT and MRI were applied in 108 (57.5%) and 80 (42.5%) patients, respectively. Positron emission tomography records were available for 61 patients. According to PET findings, 42 patients had the disease confined to the liver, while 19 patients had metastatic HCCs. Both liver parenchymal and lesion biopsies were available for 116 (61.7%) patients.

Out of 188 HCC patients, 42 patients (22.3%) had non-cirrhotic livers and cirrhosis was present in 146 (77.7%) patients. Etiology was dominantly hepatitis B and C for cirrhotic HCC patients. Fifty-three (74.7%) cirrhotic patients with HBV were treated with potent anti-viral drugs, while 9 patients were treated with either lamivudine or telbivudine. No treatment was available at the time of HCC diagnosis for 9 patients. Median time from HBV infection to diagnosis of HCC was 60 (3-135) months. Available pretreatment biopsies revealed F1-4 fibrosis for 5 (7%) patients, while 48 (67.6%) patients had pre-cirrhosis or cirrhosis on histology. Median HBV treatment duration was 12 (1-48) months. Median HBV DNA level was 127 (0-227989) IU/mL and HBV DNA levels were < 50 IU/mL for 31 (43.6%) patients at the time of HCC diagnosis. Among 35 patients with chronic liver disease secondary to HCV, 8 patients were HCV RNA negative, and 16 patients were HCVRNA positive at the time of HCC diagnosis. Three patients were treated with direct-acting anti-viral agents, while others were treated with interferon-based treatments.

No definitive underlying etiology was documented for 47.6% of non-cirrhotic, and 20.5% of cirrhotic HCC patients (*P* = .002). The rate of non-alcoholic liver disease (NAFLD) is not known definitively for patients with cirrhosis since the diagnosis of NAFLD is not easy to define precisely after the development of cirrhosis. Among 30 cryptogenic cirrhotic HCC patients, 22 patients had a history of diabetes, and 14 out of 30 patients fulfilled the metabolic syndrome criteria. Only 5 patients had radiologically or pathologically documented steatosis in the past medical records. Out of 20 non-cirrhotic HCC patients with an unknown etiology, 2 patients had grade 1 steatosis radiologically, and 6 patients were diabetic with only 1 patient fulfilling the metabolic syndrome criteria.

Among cirrhotic patients, 85 patients (58.2%) were in CPT-A stage. Forty-seven (32.2%) and 14 (9.6%) patients were in CPT-B and C stage, respectively. Platelet, INR, albumin, total bilirubin, and white blood cell levels, which reflect liver functions, differed significantly between HCC subjects with and without cirrhosis (*P* < .05). Basal demographics, clinical characteristics, and laboratory parameters of patients according to study groups are shown in Table 1.

### Tumoral Characteristics of Non-cirrhotic and Cirrhotic Hepatocellular Carcinoma Patients

Median total and largest tumor diameters were significantly larger in HCC patients with no cirrhosis (*P* = .007 and *P* < .0001, respectively). Unifocal lesion was more common in patients without cirrhosis (78.6% vs. 59.6%) (*P* = .020). Five (11.9%) patients in the non-cirrhotic and 35 (24%) patients in cirrhotic HCC groups had largest tumors less than 30 mm in diameter, while 29 (69%) patients in the non-cirrhotic and 67 (45.9%) patients in cirrhotic HCC groups had largest tumors more than 50 mm in diameter (*P* = .030). Radiological characteristics of HCC lesions did not differ between study groups, with the exception of the presence of varices and patent umbilical vein more commonly among cirrhotic patients. Compared to cirrhotic patients, histological confirmation of diagnosis with lesion biopsy was more frequently applied for non-cirrhotic HCC patients (*P* = .028). Pathological differentiation level was similar between groups. Similar percentages of patients were diagnosed within Milan and UCSF in each group. BCLC stages at the time of diagnosis were similar for both groups. Fourteen (33.4%) patients in the non-cirrhotic and 56 (38.4%) patients in cirrhotic HCC groups were detected in early BCLC stages (BCLC 0-A), and thus were candidates for curative treatments (*P* = .273) (Table 2).

### Treatment Modalities for Hepatocellular Carcinoma Patients

Treatment modalities offered to both groups of HCC patients are shown in Table 3. Surgery including resection and transplantation was applied to significantly more patients in the non-cirrhotic group (31% vs. 15.8% for non-cirrhotic and cirrhotic patients, respectively, *P* = .017). Application of other treatment modalities did not differ between the 2 groups. Curative treatment modalities including surgery (in the form of curative resection and transplantation) and RFA were applied to 15 (35.7%) non-cirrhotic and 39 (26.7%) cirrhotic HCC patients. Palliative treatments were applied to 16 (38.1%) and 48 (32.9%) of non-cirrhotic and cirrhotic HCC patients, respectively. Remaining patients were followed with best supportive care (*P* = .186).

### Survival

**Figure 1.**
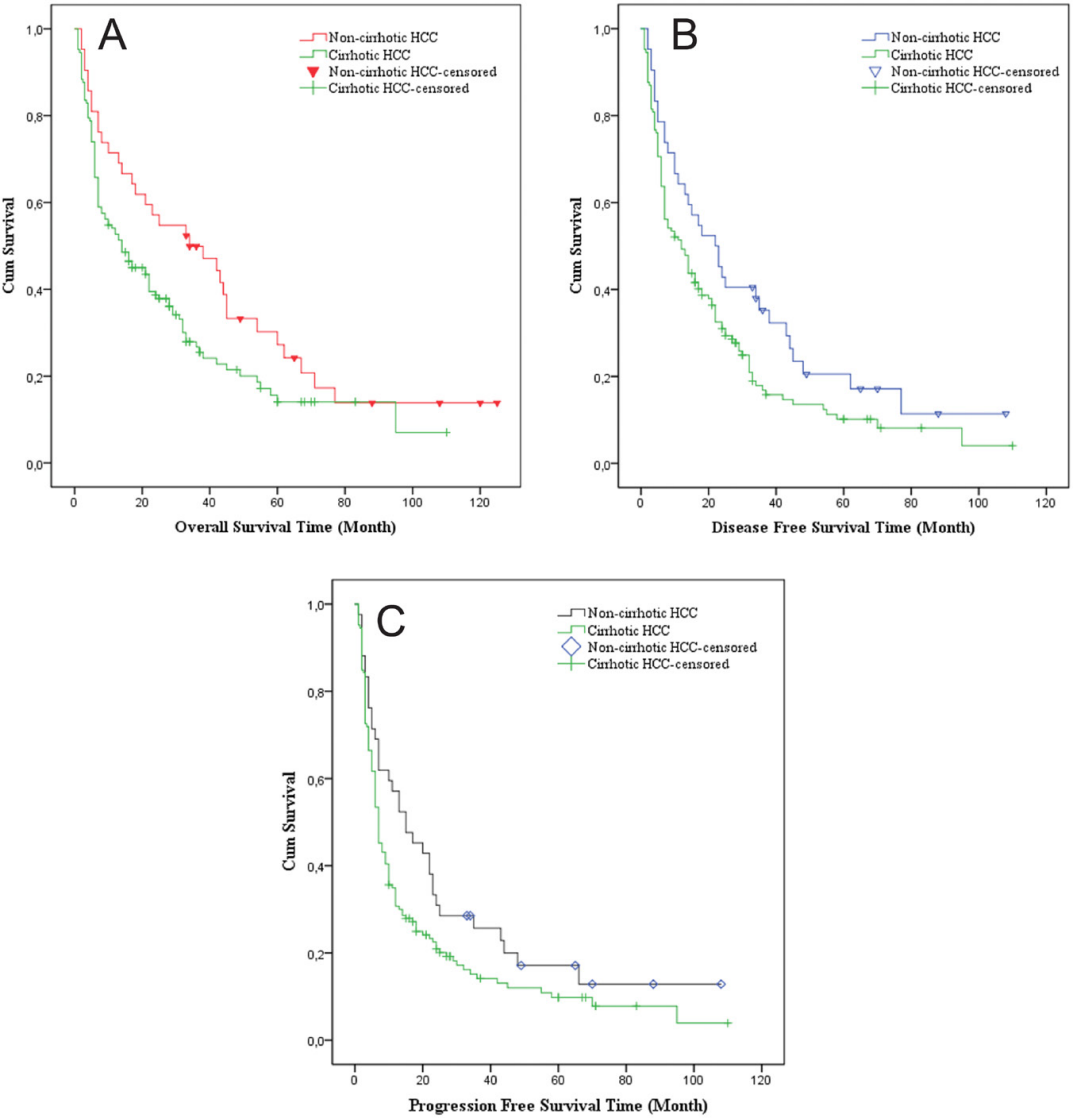
(A). Overall survival curves of HCC patients with and without underlying cirrhosis (*P* = .035), (B) Disease-free survival curves of HCC patients with and without underlying cirrhosis (*P* = .039), (C) Progression-free survival curves of HCC patients with and without underlying cirrhosis (*P* = .047). HCC, hepatocellular carcinoma.

Mean and median OS, DFS, and PFS for cirrhotic and non-cirrhotic HCC groups are shown in Table 4. Overall, disease-free, and progression-free survivals of HCC patients without cirrhosis were significantly better than those with cirrhosis (*P* < .05) as shown in Figure 1.

In univariate analysis, OS of all HCC patients did not differ with age, gender, etiology, and tumor number (*P* > .05). Total and maximal tumor diameter, low ECOG score, tumor within Milan and/or UCSF, early BCLC stage of 0-A, presence of necrosis, varices, ascites, portal vein thrombosis (PVT), vascular invasion and/or satellite lesions, pathological differentiation type, and use of curative treatment options were found to be associated with significantly better OS in all HCC patients (*P* < .005) in univariate analysis. Hepatocellular carcinoma patients diagnosed within Milan, UCSF, and early BCLC (0-A) stages had significantly better overall, disease-free, and PFS rates (*P* < .0001). Pathologic type of differentiation was found to be associated with better OS (50.0 ± 6.9 vs. 25.3 ± 5.4 months for well and poor differentiation types, respectively, *P* = .020), but not with DFS (*P* = .216) and PFS (*P* = .064) for all HCC patients.

Cox multivariate regression model revealed presence of cirrhosis (hazard ratio (HR): 2.31, 95% CI 1.47-3.62, *P* < .001), ECOG performance score (0-1 vs. 2-4) (HR: 2.12, 95% CI 1.68-2.69, *P* < .001), presence of necrosis (HR: 1.72, 95% CI 1.17-2.53, *P* = .006) and treatment modality (HR: 0.27, 95% CI 0.15-0.47, *P* < .001) as independent predictors of OS.

The factors associated with survival for non-cirrhotic HCCs were total and maximal tumor sizes (*P* < .0001), PVT (*P* = .001), ECOG performance score (0-1 vs. ≥2) (*P* = .015), pathological differentiation type (*P* = .049), BCLC stage early vs. advanced (*P* = .011), UCSF status (*P* = .006), vascular invasion (*P* = .008), presence of ascites (*P* = .021), and curative treatment modalities (*P* = .001), as seen in univariate analysis (Table 5). In Cox multivariate regression model of non-cirrhotic HCC group, curative treatment modality (HR: 0.31, 95% CI 0.11-0.87, *P* = .027) was an independent predictor for OS. Although not statistically significant, there was a trend for tumor number (HR: 1.27, 95% CI 1.00-1.63, *P* = .050) to predict OS in non-cirrhotic HCCs. Vascular invasion was the only independent predictor for DFS (HR: 2.62, 95% CI 1.01-6.93, *P* = .049) for non-cirrhotic HCCs (Table 5).

In univariate analysis of cirrhotic HCCs, total and maximal tumor sizes, ECOG performance score (0-1 vs. ≥2), PVT, CPT stage, being within Milan, UCSF and/or early BCLC stages, vascular invasion, necrosis, and infiltrative type (*P* < .0001 for all), ascites (*P* = .002), lymphadenopathy (*P* = .003), and curative treatment strategies (*P* < .0001) were found to be associated with survival (Table 5). Cox multivariate regression analysis of HCC patients with cirrhosis revealed ECOG performance score (0-1 vs. 2-4) (HR: 4.05, 95% CI 2.36-6.96, *P* < .001) and curative treatment modality (HR: 0.21, 95% CI 0.11-0.41, *P* < .001) as independent predictors for OS. Independent predictors of DFS and PFS were ECOG performance score (0-1 vs. 2-4) (HR: 4.69, 95% CI 2.75-8.00, *P* < .001 and HR: 3.14, 95% CI 1.89-5.20, *P* < .001, respectively), vascular invasion (HR: 1.63, 95% CI 1.00-2.66, *P* = .047 and HR: 1.88, 95% CI 1.18-2.99, *P* = .007, respectively), and curative treatment modality (HR: 0.39, 95% CI 0.22-0.68, *P* = .001 and HR: 0.36, 95% CI 0.20-0.64, *P* < .001, respectively) for cirrhotic HCC patients (Table 5).

## Discussion

In our study, 22.3% of all HCCs developed in non-cirrhotic patients. Liver Imaging Reporting and Data System 5 criteria were met with equal frequency within both groups. Compared to cirrhotic HCCs, HCCs in non-cirrhotic liver had larger median tumor size, but similar pathological differentiation, age, and gender distribution. Non-cirrhotic and cirrhotic HCCs were diagnosed within similar Milan and BCLC stages. Despite this, curative treatments were applied to non-cirrhotic HCC patients more frequently. Overall, disease-free and progression-free survival rates were significantly better for non-cirrhotic HCCs compared to cirrhotic HCCs. In Cox multivariate regression model, absence of cirrhosis, low ECOG performance score, absence of necrosis, and applications of curative treatment modality were predictors of longer survival for HCC patients. In Cox multivariate regression analysis, revealed application of curative treatment was associated with OS in non-cirrhotic HCC patients. Vascular invasion (*P* = .049) was predictive for DFS in non-cirrhotic HCCs as seen in multivariate analysis

Non-cirrhotic HCC rate of 22.3% in our study is higher than that reported as 11.2% in a Turkish population in which histological diagnosis rate was very low for non-cirrhotic patients.^20^ In a recent study from Türkiye including 1802 HCC patients from 14 centers, non-cirrhotic HCCs constituted 18.5% of the cohort.^21^ In a cohort by Pinyopornpanish et al,^22^ 13% of HCC patients had no liver cirrhosis. That cohort included mainly HCV and alcohol-related cirrhosis patients, and HBV was the etiological agent in only 8.3% of non-cirrhotic patients. Our study included mainly HCV and HBV related cirrhotic patients, and 42.9% of non-cirrhotic patients had HBV positivity. In accordance with our results, 2 studies from the Netherlands and Germany reported non-cirrhotic HCC rates of 19% and 19.4%, respectively.^23,24^ In a large HCC database including 1332 patients from several collaborating institutions in Türkiye, 18.6% of HCCs had no underlying cirrhosis.^25^

In our study, both groups had similar gender distribution and median age. Similar male/female ratio has been reported by Shim et al.^26^ Different from our findings, female predominance has been reported for non-cirrhotic HCCs.^10,23^ A male to female ratio of 2.7 : 1 has also been reported in a German study for non-cirrhotic HCCs.^12^ Some studies in literature reported higher median ages for non-cirrhotic HCCs.^10,12,26,27^ Etiological and geographical differences may account for these disparities. The high rate of viral etiology in our non-cirrhotic HCC group is quite different from other studies.^10,12^

In our study, non-cirrhotic HCCs were larger in diameter compared to cirrhotic HCCs. This finding is compatible with the literature.^20,26^ Similarly, in a report by van Meer et al,^23^ HCCs in non-cirrhotic livers were of similar ages to cirrhotic HCCs, more commonly unifocal, larger, likely to be candidates for curative treatment modalities, and associated with better overall survival.

Barcelona clinic liver cancer stages at diagnosis were similar for both groups in our study. In the literature, non-cirrhotic HCCs were reported to be larger and diagnosed at more advanced BCLC stages.^11,15,20,22,23^ Lack of surveillance in the non-cirrhotic liver is the reason for diagnosing HCC in a more advanced stage with a larger diameter. Hepatocellular carcinoma surveillance is recommended in the presence of cirrhosis by the liver society’s guidelines.^28,29^ 33.4% and 38.4% of non-cirrhotic and cirrhotic HCCs, respectively, were diagnosed in BCLC stage 0-A in our study. These rates were 16.3% vs. 34.9% in the study by Demirtaş et al,^20^ and 20.7% vs. 39.2% in another study from US.^22^ Although our rate for cirrhotic patients was similar to those studies, more non-cirrhotic HCCs in our study were diagnosed in early BCLC stages (0-A). Extensive use of CT and MRI, high rate of HBV as etiology in our non-cirrhotic HCC group, and our high tendency to take both parenchymal and lesion biopsies in our center from HCC patients not having radiological and clinical findings supporting cirrhosis might be reasons for diagnosing non-cirrhotic HCCs in early BCLC stages.

Hepatocellular carcinoma in non-cirrhotic liver was associated with better overall, DF and PF survival rates compared to cirrhotic HCCs in our study. Compared to cirrhotic HCCs, despite larger tumors and advanced tumor stages, better survival rates have been reported for non-cirrhotic HCCs.^10,22,23,30^ Some studies did not report a survival benefit in non-cirrhotic HCCs.^20,26^ Hepatocellular carcinoma patients who underwent resection or transplantation were included in one of those studies.^26^ Absence of cirrhosis and application of curative treatment modalities predicted better OS in our HCC cohort. The survival benefit of non-cirrhotic HCCs is mostly related to preserved liver functions which enable us to apply curative treatment strategies. Although not statistically significant, there was a trend for tumor number in non-cirrhotic HCCs to predict OS, probably by representing less tumor burden. ECOG performance score, AFP level, vascular invasion, maximal tumor diameter, and application of curative treatment modalities have been reported to influence OS in non-cirrhotic HCCs.^20,31,6^ In our study, vascular invasion was an independent predictor of DFS in non-cirrhotic HCC patients.

Absence of cirrhosis, and low ECOG performance score has been shown to be associated with OS in our HCC patients. These factors are important in the application of curative treatment strategies. For HCCs without cirrhosis, diagnosing the patient not beyond the point that makes use of curative treatments impossible, such as distant metastases or vascular invasion is important. Although NAFLD is a growing etiology of chronic liver disease, current guidelines of social associations do not recommend HCC surveillance in non-cirrhotic NAFLD patients.^28,29^ According to literature, up to one-third of HCCs from NAFLD develop in the absence of cirrhosis.^32-34^ Given the extremely large population of NAFLD, routine HCC surveillance in this population is not feasible or cost-effective. Since patients with advanced fibrosis have the greatest risk of developing HCC, NAFLD patients should be first risk stratified for this feature by validated tools to justify HCC surveillance. The American Gastroenterology Association has recommended that NAFLD patients with documented advanced fibrosis by non-invasive methods should be considered for HCC surveillance.^35^ From this point of view, non-cirrhotic NAFLD patients, and similarly HCV patients with a sustained virological response, having a significant degree of fibrosis should be more closely followed up. Further studies are needed to differentiate metabolic or genetic risk factors for HCC development in these patients to develop surveillance strategies.

Advantage of this study is high histological diagnosis rate especially in non-cirrhotic HCC patients. A study by Paradis et al^36^ has shown development of well-differentiated HCCs more frequently in the presence of low fibrosis (75% in F0-F2 vs. 45% in F3-F4 fibrosis). As a potential bias, only surgically managed HCCs were included in this analysis. Hepatocellular carcinoma associated with NAFLD or metabolic syndrome have been reported to be often moderately or well differentiated.^37,38^ In our study, 76.2% of non-cirrhotic HCCs had a histological diagnosis whatever the treatment. Pathological differentiation did not differ between groups. Well-differentiated tumors comprised 45.5% of all non-cirrhotic HCCs, while 38.9 % of cirrhotic HCCs were well-differentiated (*P* = .866). Etiology was HBV in 42.9% of our non-cirrhotic patients. Of note, HBV has direct oncogenic potential as a result of HBV DNA integration into the hepatocyte genome.^39^ Etiological differences in different studies could account for differentiation levels of HCCs in non-cirrhotic livers.

Major limitations for this study are the relatively low number of patients, and its retrospective nature that prevented us from presenting some data, for example, body-mass index and metabolic parameters. This study also represents findings from a single tertiary center. Some patients were referred to our institution for further treatment after the initial diagnosis in another center. This could partly account for late stages of diagnosis in some part of patients. Another limitation is the relatively high rate of viral hepatitis among patients which prevents us from generalizing the findings especially to non-alcoholic steatohepatitis (NASH)-related HCCs.

In conclusion, this study is one of the few studies presenting characteristics and survival of cirrhotic and non-cirrhotic HCCs in the Turkish population. Our study showed the diagnosis of non-cirrhotic HCCs in larger tumor size, but with similar ages, tumor stage, and pathological differentiation level compared to cirrhotic HCCs. Hepatocellular carcinomas in non-cirrhotic liver were associated with better survival rates.

## Figures and Tables

**Table 1.** Demographic and Laboratory Findings of all Hepatocellular Carcinoma Patients With and Without Underlying Cirrhosis

	Non‐cirrhotic (n = 42)	Cirrhotic (n = 146)	Total (n = 188)	*P*
Sex (male) n (%)	38 (90.5)	116 (79.5)	154 (81.9)	.102
Age [median (IQR)]	64 (26-92)	64 (44-84)	64 (26-92)	.208
Age groups n (%)				**.004**
	0-54	11 (26.2)	14 (9.6)		25 (13.3)
	55-64	10 (23.8)	61 (41.8)		71 (37.8)
	65-74	10 (23.8)	50 (34.2)		60 (31.9)
75+	11 (26.2)	21 (14.4)	32 (17.0)	
Alcohol				
n (%)	5 (11.9)	25 (17.1)	30 (16.0)	.416
Etiology n (%)				**.002**
	HBV	18 (42.9)	71 (48.6)		89 (47.3)
	HCV	4 (9.5)	35 (24.0)		39 (20.7)
	Unknown	20 (47.6)	30 (20.5)		50 (26.6)
Alcohol	0 (.0)	10 (6.8)	10 (5.3)	
Diabetes n (%)	14 (33.3)	61 (41.8)	75 (39.9)	.15
T. bilirubin (mg/dL)*	0,7 (0.5-1.0)	1.2 (0.8-2.0)	1.1 (0.7-1.8)	**.001**
Albumin*	4.0 (3.6-4.3)	3.5 (3.1-3.9)	3.6 (3.2-4.0)	**.001**
AST*	52.5 (26.0-81.0)	53 (37.0-101.0)	53 (37.0-95.0)	.364
ALT*	47.5 (24.0-84.0)	35 (25.0-62.0)	36.5 (25.0-66.0)	.346
INR*	1.1 (1.0-1.2)	1.2 (1.1-1.3)	1.2 (1.1-1.3)	**.001**
Creatinine (mg/dL)*	0.8 (0.7-1.0)	0.8 (0.7-1.0)	0.8 (0.7-1.0)	.764
Hb*	13.2 (11.4-14.7)	12.4 (11.2-13.7)	12.6 (11.3-13.9)	.122
WBC*	7.8 (6.0-8.9)	5.9 (4.69-8.40)	6.45 (4.80-8.50)	**.005**
Platelet*	261 500 (204 000-353 000)	135 500 (940 00-192 000)	154 000 (100 500-228 000)	**.001**
CRP*	34.2 (14.6-63.1)	30.1 (7.9-56.1)	31.5 (10.6-57.9)	.149
AFP*	18.8 (4.2-450.0)	43.3 (5.4-589.1)	40.1 (5.1-574.4)	.400
AFP >10 ng/mL n (%)	25 (59.5)	97 (66.4)	122 (64.9)	.72
MELD	N/A	9 (8.0-13.0)		
Child stage				
		A			N/A	85 (58.2)
		B					47 (32.2)
C		14 (9.6)			
ECOG-PS				.282
	0	17 (40.5)	40 (27.4)		57 (30.3)
	1	16 (38.1)	76 (52.1)		92 (48.9)
	2	7 (16.7)	19 (13.0)		26 (13.8)
3	2 (4.8)	11 (7.5)	13 (6.9)	

Values in bold indicate statistical signifcance.

AFP, alpha-fetoprotein; ALT, alanine aminotransferase; AST, aspartate aminotransferase; CRP, C-reactive protein; ECOG-PS, Eastern Cooperative Oncology Group performance status; HBV, hepatitis B virus;, HCV, hepatitis C virus; INR, international normalized ratio; MELD, Model for End-stage Liver Disease; WBC, white blood cell.

*Median (interquartile range).

**Table 2.** Tumor-Related Characteristics in Hepatocellular Carcinoma Patients With and Without Underlying Cirrhosis

	Non‐Cirrhotic (n = 42)	Cirrhotic (n = 146)	Total (n = 188)	*P*
Biopsy n (%)	32 (76.2)	84 (57.5)	116 (61.7)	**.028**
Biopsy type n (%)				.159
				True-cut biopsy	22 (68.8)	65 (77.4)	87 (75.0)	
				Surgical resection	10 (31.3)	15 (17.9)	25 (21.6)	
Total hepatectomy	0 (.0)	4 (4.8)	4 (3.4)	.866
Differentiation n (%)				.866
					Well	10 (45.5)	21 (38.9)	31 (40.8)
					Moderate	6 (27.3)	16 (29.6)	22 (28.9)
Poor	6 (27.3)	17 (31.5)	23 (30.3)	
Within MILAN n (%)	12 (28.6)	58 (39.7)	70 (37.2)	.188
BCLC stage n (%)				.273
					BCLC-0	2 (4.8)	9 (6.2)	11 (5.9)
					BCLC-A	12 (28.6)	47 (32.2)	59 (31.4)
					BCLC-B	8 (19.0)	21 (144)	29 (15.4)
					BCLC-C	20 (47.6)	56 (38.4)	76 (40.4)
BCLC-D	0 (.0)	13 (8.9)	13 (6.9)	
Within UCSF n (%)	15 (35.7)	76 (52.1)	91 (48.4)	.062
Number of tumors				**.020**
					1	33 (78.6)	87 (59.6)	120 (63.8)
					2	1 (2.4)	26 (17.8)	27 (14.4)
3+	8 (19)	33 (22.6)	41 (21.8)	
Total tumor size*	89.5 (16-240)	59.0 (12-290)	67.0 (12-290)	**.007**
Total tumor size (mm)				.403
					<20	1 (2.4)	7 (4.8)	8 (4.3)
					20-29	4 (9.5)	20 (13.7)	24 (12.8)
					30-65	11 (26.2)	50 (34.2)	61 (32.4)
>65	26 (61.9)	69 (47.3)	95 (50.5)	
Largest tumor size*	81.5 (16-182)	46 (12-160)	52 (12-182)	**<.0001**
Largest tumor size (mm)				**.030**
					<20	1 (2.4)	7 (4.8)	8 (4.3)
					20-29	4 (9.5)	28 (19.2)	32 (17.0)
					30-49	8 (19.0)	44 (30.1)	52 (27.7)
>50	29 (69.0)	67 (45.9)	96 (51.1)	
Varices	0 (.0)	63 (43.2)	63 (33.5)	**.001**
PVT	10 (23.8)	50 (34.2)	60 (31.9)	.201
Vascular invasion	20 (47.6)	53 (36.3)	73 (38.8)	.185
Necrosis	23 (54.8)	60 (41.1)	83 (44.1)	.116
Infiltrative type	12 (28.6)	38 (26.0)	50 (26.6)	.742
Arterial contrast	33 (78.6)	130 (89.0)	163 (86.7)	.078
Washout	36 (85.7)	130 (89.0)	166 (88.3)	.554
Capsule enhancement	21 (50.0)	61 (41.8)	82 (43.6)	.344
LAP	3 (7.1)	13 (8.9)	16 (8.5)	.718
Patent umbilical vein	3 (7.1)	34 (23.3)	37 (19.7)	**.020**
Satellite lesions	10 (23.8)	38 (26.0)	48 (25.5)	.771

Values in bold indicate statistical signifcance.

BCLC, Barcelona clinic liver cancer; HCC, hepatocellular carcinoma; LAP, lymphadenopathy; PVT, portal vein thrombosis; BCLC, Barcelona clinic liver cancer, UCSF, University of California San Francisco.

*Median (interquartile range).

**Table 3.** Treatment Modalities for Groups

	Non‐cirrhotic (n = 42)		Cirrhotic (n = 146)		Total (n = 188)		
	n	%	n	%	n	%	*P*
Immunotherapy							.750
	No	39	(95.1)	128	(96.2)	167		(96.0)
Yes	2	(4.9)	5	(3.8)	7	(4.0)	
Surgery							**.017**
	No	29	(69.0)	123	(84.2)	152		(80.9)
	Resection	12	(28.6)	16	(11.0)	28		(14.9)
Transplantation	1	(2.4)	7	(4.8)	8	(4.3)	
Chemotherapy							.524
	No	37	(90.2)	124	(93.2)	161		(92.5)
Yes	4	(9.8)	9	(6.8)	13	(7.5)	
RFA							.448
	No	36	(87.8)	111	(82.8)	147		(84.0)
Yes	5	(12.2)	23	(17.2)	28	(16.0)	
Sorafenib							.690
	No	30	(73.2)	93	(69.9)	123		(70.7)
Yes	11	(26.8)	40	(30.1)	51	(29.3)	
TACE							.650
	No	27	(64.3)	97	(72.4)	124		(70.5)
	1 session	9	(21.4)	18	(13.4)	27		(15.3)
	2 sessions	3	(7.1)	10	(7.5)	13		(7.4)
3 sessions	3	(7.1)	9	(6.7)	12	(6.8)	
TARE							.695
	No	38	(92.7)	118	(88.7)	156		(89.7)
	1 session	2	(4.9)	12	(9.0)	14		(8.0)
2 sessions	1	(2.4)	3	(2.3)	4	(2.3)	

Value in bold indicates statistical signifcance.

fRFA, radiofrequency ablation, TACE, transarterial chemoembolization, TARE, transarterial radioembolization.

**Table 4.** Mean and Median Survival of Patients With and Without Underlying Cirrhosis

	OS		DFS		PFS	
	Non‐cirrhotic (n = 42)	Cirrhotic (n = 146)	Non‐cirrhotic (n = 42)	Cirrhotic (n = 146)	Non‐cirrhotic (n = 42)	Cirrhotic (n = 146)
Mean (median) months	43.3 (34)	29.2 (14)	33.7 (22)	23.3 (12)	29.1 (15)	19.3 (7)
*P*	.035		.039		.047	
12 months	71.4%	54.0%	64.3%	51.2%	57.1%	34.7%
24 months	57.1%	39.4%	45.2%	32.5%	33.3%	22.3%
36 months	49.7%	28.3%	35.2%	19.5%	25.6%	16.2%
48 months	33.6%	21.8%	23.9%	15.0%	19.9%	13.0%
60 months	30.7%	16.0%	20.7%	11.6%	16.9%	10.9%

DFS, disease-free survival; OS, overall survival; PFS, progression-free survival.

**Table 5.** Results of Univariate and Multivariate Analyses for Overall, Disease-Free, and Progression-Free Survival in Patients With Cirrhotic and Non-Cirrhotic Hepatocellular Carcinoma

	Non-Ccirrhotic HCC				Cirrhotic HCC			
	**Univariate *P***	*P*	HR	95% CI	**Univariate *P***	*P*	HR	95% CI
Overall survival								
Tumor number	.008	.050	1.27	1.00-1.63	.631	.927	0.99	0.87-1.13
ECOG-PS	.015	.644	0.74	0.22-0.54	<.001	<.001	4.05	2.36-6.96
PVT	.001	.159	3.20	0.63-16.17	<.001	.119	1.49	0.90-2.47
Vascular invasion	.008	.515	1.36	0.53-3.50	<.001	.121	1.48	0.90-2.42
No treatment	.001		Ref		<.001		Ref	
Treatment (palliative)		.397	0.65	0.25-1.73		.001	0.44	0.27-0.71
Treatment (curative)		.027	0.31	0.11-0.87		<.001	0.21	0.11-0.41
Disease-free survival								
Tumor number	.087	.145	1.19	0.94-1.52	.461	.711	0.97	0.86-1.10
ECOG-PS	.001	.617	1.32	0.44-3.96	<.001	<.001	4.69	2.75-8.00
PVT	<.001	.165	2.68	0.66-10.83	<.001	.196	1.37	0.85-2.21
Vascular invasion	.003	.049	2.62	1.01-6.93	<.001	.047	1.63	1.00-2.66
No treatment	<.001		Ref		<.001		Ref	
Treatment (palliative)		.890	0.93	0.37-2.35		.002	0.49	0.31-0.77
Treatment (curative)		.229	0.51	0.17-1.51		.001	0.39	0.22-0.68
Progression-free survival								
Tumor number	.129	.495	1.08	0.86-1.36	.328	.822	1.01	0.89-1.14
ECOG-PS	.010	.962	1.02	0.36-2.86	<.001	<.001	3.14	1.89-5.20
PVT	<.001	.219	2.22	0.62-7.98	<.001	.106	1.44	0.92-2.27
Vascular invasion	.038	.111	2.10	0.86-5.25	<.001	.007	1.88	1.18-2.99
No treatment			Ref				Ref	
Treatment (palliative)		.775	1.14	0.45-2.91		.007	0.54	0.34-0.84
Treatment (curative)	.003	.150	0.46	0.16-1.32	<.001	<.001	0.36	0.20-0.64

ECOG-PS, Eastern Cooperative Oncology Group performance status; HCC, hepatocellular carcinoma; PVT, portal vein thrombosis.
